# Disability Among Ebola Survivors and Their Close Contacts in Sierra Leone: A Retrospective Case-Controlled Cohort Study

**DOI:** 10.1093/cid/cix705

**Published:** 2017-08-20

**Authors:** Soushieta Jagadesh, Stephen Sevalie, Richard Fatoma, Foday Sesay, Foday Sahr, Brian Faragher, Malcolm G Semple, Tom E Fletcher, Ralf Weigel, Janet T Scott

**Affiliations:** 1 Liverpool School of Tropical Medicine, United Kingdom; 2 34 Military Hospital, Freetown, Sierra Leone; 3 University of Liverpool Institute of Translational Medicine and National Institute for Health Research Health Protection Research Unit in Emerging and Zoonotic Infections, United Kingdom; 4 Department of Infectious Diseases and Clinical Microbiology, Ondokuz Mayis University Hospital, Samsun, Turkey

**Keywords:** disability, Ebola survivors, postinfection disability, Washington Group Disability Questionnaire, EVD convalescence

## Abstract

Ebola survivors (21/27 [77.8%]) suffered more disability than their close contacts (6/54 [11.1%]) (adjusted odds ratio, 23.5 [95% confidence interval, 6.5–85.7]; *P* < .001) when measured by the Washington Group Disability Extended Questionnaire. Major limitations in vision, mobility, cognition, and affect were observed in survivors 1 year following the 2014–2016 Ebola outbreak, highlighting the need for long-term rehabilitation.

The scale of the 2014–2016 West African Ebola outbreak has resulted in an unprecedented number of survivors and the opportunity to vastly improve the understanding of the health challenges they face [1]. In early convalescence from Ebola virus disease (EVD), ocular, musculoskeletal, and neuropsychiatric sequelae are common [2–5]. Reports from the Bundibuygo and Kikwit outbreaks suggest that there are also long-term complications [6, 7]. Difficulties of survivors in resuming work after EVD were reported following the Gulu outbreak in Uganda [8].

We assessed disability among a cohort of EVD survivors 12 months following their discharge and compared with their close contacts in Freetown, Sierra Leone.

## METHODS

We recruited study participants in June 2016 by systematic sampling from a list of attendees at the Ebola Survivors Clinic, 34th Military Hospital in Freetown, Sierra Leone. Based on background disability surveys from Sierra Leone [9] and data from EVD cross-sectional studies, 50% of survivors were estimated to have 1 or more forms of disability compared with 17% in the general population. A total sample size of 81 (27 EVD survivors and 54 contacts) was estimated as being required to detect a difference of this magnitude or greater with 80% power using a conventional 2-sided significance level of 5% (GPower 3.1, formula based on Fisher exact test). Inclusion criteria for EVD survivors were confirmed EVD by polymerase chain reaction testing, age over 19 years, completion of ≥12 months of convalescence at the time of recruitment, and verification of an EVD discharge certificate. Each EVD survivor recruited was requested to bring 2 close contacts (1:2) from during the time of disease, preferably members of their family, who had never been admitted to an Ebola treatment unit and were not enrolled in an EVD vaccine trial. All close contacts recruited as controls were from the same community as cases at time of disease, same language group, and similar socioeconomic status as the cases.

Disability was measured using the Washington Group Disability Extended Questionnaire for both the EVD survivors and their nonaffected contacts. The questionnaire measured self-reported physical and mental impairments present at the time of the interview (Supplementary Materials). The questionnaire assesses 6 domains: vision, hearing, mobility, self-care, communication, and cognition. Functionality scores were calculated from the severity and frequency of anxiety, depression, pain, and fatigability. We conducted face-to-face interviews in Krio and English and recorded the responses in an electronic format of the questionnaire.

Categorical disability measures were summarized using frequency counts and percentages; differences between the exposed (survivor) and unaffected contacts (control) subjects were summarized as odds ratios (ORs) with their exact (binomial) 95% confidence intervals (CIs), after adjustment for age and sex using logistic regression (aOR). Continuous disability measures were summarized using means and standard deviations; differences between the exposed and unexposed subjects were summarized as mean differences with their 95% CIs, after adjustment for age, sex, and occupation using linear regression. The other demographic factors (including place of residence) did not contribute as confounding factors during statistical modeling. All statistical tests were 2 tailed, with significance set at the conventional 5% level. All analyses were done using Stata software version 14.

All participants provided written informed consent. The protocol was approved by the institutional ethics review board of The Liverpool School of Tropical Medicine, United Kingdom (10 May 2016), and the Sierra Leone Ethics and Scientific Review committee (31 May 2016).

## RESULTS

Twenty-seven EVD survivors (cases) and 54 unaffected contacts (controls) were recruited. The EVD survivors were more likely to be >25 years of age (n = 21 [77.8%]) than the controls (29 [53.7%]) and to be female (n = 21 [77.8%] vs 29 [53.7%]) (Fisher exact test *P* = .05 for both). At the time of the study, EVD survivors were less likely to live in the Western Urban area, outside Freetown (n = 11 [40.7%] vs 36 [66.7%]: Fisher exact test *P* = .03) and more likely to be unemployed due to health reasons (n = 4 [14.8%] vs 0; Fisher exact test *P* = .01). There was no significant difference in preexisting comorbidities between the 2 groups at a median time of 18 months postdischarge.

Disability in at least 1 of the 6 functional domains was reported by significantly more EVD survivors than controls (aOR, 23.5 [95% CI, 6.5–85.7]) (Table 1). EVD survivors had higher odds of blurred vision (aOR, 7.6 [2.0–27.9]). Subjective hearing loss was observed (OR, 12.05 [95% CI, 1.31–110.6]; *P* = .03), but this was not statistically significant when adjusted for age (aOR, 11.5 [95% CI, .6 – 214]; *P* = .1). Differences in physical disability were most marked with the survivors’ cohort being more likely to experience difficulty in walking 100 m, 500 m, climbing 12 stairs, and “moderate disability in mobility” than controls (aOR for each ranging from 64 to 206; all *P* < .001).

**Table 1.** Comparison of Disability Between the Ebola Virus Disease Survivors (Cases) and Unaffected Contacts (Controls)

Comorbities and Limitations Measured	Cases	Controls	Unadjusted OR (95% CI)	*P* Value	Adjusted OR (95% CI)	*P* Value
Sample size	27	54				
Preexisting comorbidities reported	4 (14.8)	3 (5.6)	2.96 (.61–14.43)	.180	1.55 (.33–7.19)^a^	.579
Some disability	21 (77.8)	6 (11.1)	17.85 (8.02–97.74)	<.001	23.52 (6.46–85.67)^a^	<.001
Disability with long-distance vision	7 (25.9)	3 (5.6)	5.95 (1.39–25.54)	.016	6.65 (1.53–28.88)^a^	.011
Disability with near-distance vision	11 (40.7)	4 (7.4)	8.59 (2.38–31.01)	.001	10.31 (2.75–38.58)^a^	.001
Blurred vision	12 (44.4)	5 (9.3)	7.84 (2.36–26.04)	.001	7.55 (2.04–27.93)^a^	.002
Subjective hearing disability	5 (18.5)	1 (1.9)	12.05 (1.31–110.6)	.028	11.47 (.62–213.8)^a^	.102
Limitations in walking 500 m	20 (74.1)	2 (3.7)	74.29 (14.07–392.3)	<.001	94.30 (11.73–757.8)^a^	<.001
Limitations in climbing 12 steps	23 (85.2)	4 (7.4)	71.87 (16.35–315.9)	<.001	64.76 (13.68–306.6)^a^	<.001
Minimal disability in mobility	23 (85.2)	4 (7.4)	71.87 (16.35–315.9)	<.001	64.76 (13.68–306.6)^a^	<.001
Moderate disability in mobility	18 (66.7)	1 (1.9)	106.0 (12.38–907.6)	<.001	205.6 (19.95–2119)^a^	<.001
Severe disability in mobility	4 (14.8)	0	NC	.011^b^	NC	
Difficulty in self-care	0	0	NC		NC	
Difficulty in communication	0	0	NC		NC	
Difficulty remembering/concentrating	9 (33.3)	0	NC	<.001^b^	NC	
Pain score, mean (SD)	4.07 (2.69)	0.89 (1.70)	3.18^c^ (2.21–4.16)	<.001	2.51^c^ (1.33–3.69)^d^	<.001
Fatigability score, mean (SD)	3.26 (2.65)	0.39 (0.76)	2.87^c^ (2.10–3.64)	<.001	2.23^c^ (1.36–3.09)^d^	<.001
Anxiety, mean (SD)	3.37 (3.49)	1.04 (1.60)	2.33^c^ (1.21–3.46)	<.001	1.89^c^ (.52–3.27)^d^	.008
Depression, mean (SD)	5.07 (4.19)	0.96 (1.35)	4.11^c^ (2.87–5.35)	<.001	3.32^c^ (1.95–2.59)^d^	<.001

Data are presented as No. (%) unless otherwise indicated.

Abbreviations: CI, confidence interval; NC, not calculable; OR, odds ratio; SD, standard deviation.

^a^Adjusted for age and sex.

^b^Fisher exact test.

^c^Mean difference in scores.

^d^Adjusted for age, sex, and occupation.

Self-rated levels of pain, fatigue, anxiety, and depression influenced disability in mobility. Relative to controls, the EVD survivors had very significantly increased mean pain scores (adjusted mean difference, 2.51 [95% CI, 1.33–3.69]), fatigue scores (2.23 [95% CI, 1.36–3.09]), anxiety scores (1.89 [95% CI, .52–3.27]), and depression scores (3.32 [95% CI, 1.95–2.59]). Mean fatigue scores were significantly higher for female than for male EVD survivors (adjusted mean difference, 3.12 [95% CI, .88–5.36]; *P* = .008) but were similar for the 2 sexes among the controls (0.05 [95% CI, –.37 to .48]; *P* = .799). No EVD survivors or contacts reported disturbances in self-care and communication.

When compared to their controls, EVD survivors had significantly higher subjective difficulties remembering or concentrating (9/27 [33.3%] vs 0; *P* < .001).

## DISCUSSION

This study provides case-controlled data on disability in EVD survivors, showing that they have higher odds of developing disability in vision, mobility, and cognition 1 year after recovery from acute disease in comparison to their contacts.

We observed that mobility limitation was the most common post-Ebola disability in EVD. The survivors reported significantly higher odds of limitations in walking 500 m and climbing stairs. Musculoskeletal pain was the major contributor to mobility limitations. Our findings reporting long-term musculoskeletal pain concur with the studies from Kikwit and Bundibuygo [6, 7].

We also observed that survivors of EVD are more likely to have blurred vision than their contacts. Ocular sequelae have been demonstrated in survivors from West Africa [4, 10] and require specialist assessment, and in the long term cataract replacement is frequently indicated. We did not observe a statistically significant difference in self-reported hearing loss between the 2 groups. The evidence from the 1995 Ebola virus outbreak shows that the post-EVD complaint of hearing loss was not significant by audiometry, 21 months following the outbreak [7]. Subjective hearing loss described in studies [2, 4] during early convalescence may recover within months as it can with Lassa fever [11].

The psychological effects of EVD are often neglected in the acute setting but would be expected to persist into convalescence and may compound physical disabilities. Our data showed that the adjusted mean difference of depression and anxiety scores were significantly higher in EVD survivors, compared to controls, and that a third of survivors also had significant difficulties with concentration. This subjective post-EVD cognitive impairment coincides with the short-term memory problems that have been reported in earlier studies [5, 12]. This may be sequelae of critical illness or suggest direct viral neurological involvement.

The main limitation of the study is dependence on self-reporting of disabilities. We mitigated this by design using a validated questionnaire with objective measurements of disability and community controls. The selection of controls (contacts not affected by EVD) by the survivors may have introduced some bias, although 22% did have evidence of disability, consistent with previous population estimates [9]. We were unable to screen serologically for asymptomatic EVD infection in the control group. However, asymptomatic EBOV infection has recently been shown to be uncommon in the Western area of Sierra Leone, even in close contacts [13]. Although adequately powered, the study sample size was small, resulting in wide confidence intervals. This study only focused on the investigation of disability in adults, whereas pediatric survivors remain an important understudied and vulnerable group. Despite these limitations, the study provides statistically significant case-controlled evidence on post-Ebola disability in EVD survivors using a standardized disability questionnaire. Displacement of survivors from their communities following the outbreak remains a concern to be addressed.

Further research in this cohort is required to understand the pathogenesis of sequelae and characterize disability further. It is clear that EVD survivors require an integrated package of care. Long-term treatment and rehabilitation strategies are challenging in the context of a constrained health system but require advocacy, investment, and a holistic approach. Specific interventions such as physiotherapy have been reported to be effective after chikungunya disease [14], and might benefit EVD survivors. Task shifting of rehabilitation services to community-based rehabilitation programs for identifying disability and accessing assistive devices may prove an effective strategy.

This study has demonstrated that a year following acute disease, survivors of the recent EVD outbreak have higher odds of persisting disability in mobility, vision, and cognition. Mental health issues such as anxiety and depression persist in EVD survivors and must not be neglected. Further evaluation of the scale of disability in larger survivor cohorts is required, as is a new focus on sustainable long-term rehabilitation in EVD survivors.

## Supplementary Data

Supplementary materials are available at *Clinical Infectious Diseases* online. Consisting of data provided by the authors to benefit the reader, the posted materials are not copyedited and are the sole responsibility of the authors, so questions or comments should be addressed to the corresponding author.

## Supplementary Material

Questionnaire_Supplement_1

## Notes

***Financial support.*** The study was part of a Master’s dissertation for the Liverpool School of Tropical Medicine and was facilitated by a wider project, “Characterising Post Ebola Syndrome, Sierra Leone” supported by the Wellcome Trust Enhancing Research Activity in Epidemic Situations (ERAES program [grants to J. T. S. and M. G. S.]). J. T. S. and M. G. S. are supported by the UK National Institute for Health Research Health Protection Research Unit in Emerging and Zoonotic Infections. T. F. is funded by the Wellcome Trust (104480/Z/14/Z) and the UK Ministry of Defence.

***Potential conflicts of interest.*** All authors: No reported conflicts of interest. All authors have submitted the ICMJE Form for Disclosure of Potential Conflicts of Interest. Conflicts that the editors consider relevant to the content of the manuscript have been disclosed.

## References

[1] World Health Organization. WHO meeting on survivors of Ebola virus disease: clinical care of survivors. Available at: http://apps.who.int/iris/bitstream/10665/204126/1/9789241509794_eng.pdf. Accessed 25 May 2016.

[2] Mattia JG, Vandy MJ, Chang JC Early clinical sequelae of Ebola virus disease in Sierra Leone: a cross-sectional study. Lancet Infect Dis 2016; 16:331–8. 26725449 10.1016/S1473-3099(15)00489-2 PMC5617647

[3] Scott JT, Sesay FR, Massaquoi TA, Idriss BR, Sahr F, Semple MG Post-Ebola syndrome, Sierra Leone. Emerg Infect Dis 2016; 22:641–6. 26983037 10.3201/eid2204.151302 PMC4806950

[4] Tiffany A, Vetter P, Mattia J Ebola virus disease complications as experienced by survivors in Sierra Leone. Clin Infect Dis 2016; 62:1360–6. 27001797 10.1093/cid/ciw158 PMC4872294

[5] Qureshi AI, Chughtai M, Loua TO Study of Ebola virus disease survivors in Guinea. Clin Infect Dis 2015; 61:1035–42. 26060289 10.1093/cid/civ453

[6] Clark DV, Kibuuka H, Millard M Long-term sequelae after Ebola virus disease in Bundibugyo, Uganda: a retrospective cohort study. Lancet Infect Dis 2015; 15:905–12. 25910637 10.1016/S1473-3099(15)70152-0

[7] Rowe AK, Bertolli J, Khan AS Clinical, virologic, and immunologic follow-up of convalescent Ebola hemorrhagic fever patients and their household contacts, Kikwit, Democratic Republic of the Congo. Commission de Lutte contre les Epidémies à Kikwit. J Infect Dis 1999; 179(suppl 1):S28–35. 9988162 10.1086/514318

[8] Wendo C Caring for the survivors of Uganda’s Ebola epidemic one year on. Lancet 2001; 358:1350. 10.1016/S0140-6736(01)06467-4 11684230

[9] Trani J, Ban O, Bailey N. Disability in and around urban areas of Sierra Leone. 2009 Available at: https://www.ucl.ac.uk/lc-ccr/downloads/Disability_in_and_Around_Urban_Areas_of_Sierra_Leone.pdf. Accessed 1 March 2016.

[10] Varkey JB, Shantha JG, Crozier I Persistence of Ebola virus in ocular fluid during convalescence. N Engl J Med 2015; 372:2423–7. 25950269 10.1056/NEJMoa1500306 PMC4547451

[11] Liao BS, Byl FM, Adour KK Audiometric comparison of Lassa fever hearing loss and idiopathic sudden hearing loss: evidence for viral cause. Otolaryngol Head Neck Surg 1992; 106:226–9. 1589210 10.1177/019459989210600303

[12] Epstein L, Wong KK, Kallen AJ, Uyeki TM Post-Ebola signs and symptoms in U.S. survivors. N Engl J Med 2015; 373:2484–6. 10.1056/NEJMc1506576 26672870

[13] Glynn JR, Bower H, Johnson S Asymptomatic infection and unrecognised Ebola virus disease in Ebola-affected households in Sierra Leone: a cross-sectional study using a new non-invasive assay for antibodies to Ebola virus. Lancet Infect Dis 2017; 17:645–53. 28256310 10.1016/S1473-3099(17)30111-1 PMC6520246

[14] Javelle E, Ribera A, Degasne I, Gaüzère BA, Marimoutou C, Simon F Specific management of post-chikungunya rheumatic disorders: a retrospective study of 159 cases in Reunion Island from 2006-2012. PLoS Negl Trop Dis 2015; 9:e0003603. 25760632 10.1371/journal.pntd.0003603 PMC4356515

