# Supplementary material for: Disability Among Ebola Survivors and Their Close Contacts in Sierra Leone: A Retrospective Case-Controlled Cohort Study
Source: Clin Infect Dis. 2017 Aug 20;66(1):131–3. doi: 10.1093/cid/cix705 (PMC5833946; doi:10.1093/cid/cix705)
Supplement: Questionnaire_Supplement_1 [file cix705_suppl_questionnaire_supplement_1.docx]

Participant Id:

Date and time of the interview: MM/DD/YYYY hh:mm AM/PM

Interview checklist

- Informed consent signed: yes/no
- Debriefing session done: yes/no
- Participant ID written on consent form and questionnaire: yes/no
- Refreshment for the candidate available: yes/no
- Participant looks: comfortable/anxious/ nervous/ uninterested
- Noise or other disturbances in the room: Very disturbing, I cannot hear the participant/ Can hear the participant but distracting/ No disturbances
- Place of survey: Outdoors/ Indoors; People often bustling in/ Indoors; Quiet private area of the room

Age: 19-24; 25-34; 35-44; 45-54; 55-64; 65-74; over 75

Sex: female/ male

Address: From Freetown/ Outside of Freetown from Western Urban area/ Outside Western Urban area but from Sierra Leone/ Outside Sierra Leone

Which describes your occupational status best?

|  |  | Paid work |  |
| --- | --- | --- | --- |
|  |  | Self-employed, such as own your business or farming |  |
|  |  | Non-paid work, such as volunteer or charity |  |
|  |  | Student |  |
|  |  | Keeping house/ homemaker |  |
|  |  | Retired |  |
|  |  | Unemployed (health reasons) |  |
|  |  | Unemployed (other reasons) |  |
|  |  | Other (specify)______________ _____________________ |  |

Duration since EVD (to be asked to survivors): in months

Have you been diagnosed with any chronic illness prior to the epidemic?

Diabetes mellitus/ systemic hypertension/ Cardiac disease/ chronic renal failure/ cerebrovascular disease or stroke/ Lung diseases like COPD or bronchial asthma/Liver disorders/ HIV/ Tuberculosis/No/ Refused/ Other

Are you currently taking any medication?

Yes/no; if yes, specify

Have you been diagnosed with Lassa fever? Yes/no

If yes, how many years ago?

History of river blindness? Yes/no

Start of CDC’s Washington Group - Extended Question Set on Functioning (WG ES-F)

*Interviewer, reads:* “Now I am going to ask you some questions about your ability to do different activities, and how you have been feeling.”

CORE

1. Do you have difficulty seeing, even if wearing glasses?

| No - no difficulty |  |
| --- | --- |
| Yes – some difficulty |  |
| Yes – a lot of difficulty |  |
| Cannot do at all |  |

1. Do you have difficulty hearing, even if using a hearing aid?

| No - no difficulty |  |
| --- | --- |
| Yes – some difficulty |  |
| Yes – a lot of difficulty |  |
| Cannot do at all |  |

1. Do you have difficulty walking or climbing steps? a. No- no difficulty

b. Yes – some difficulty c. Yes – a lot of difficulty d. Cannot do at all

1. Do you have difficulty remembering or concentrating? a. No – no difficulty

b. Yes – some difficulty c. Yes – a lot of difficulty d. Cannot do at all

1. Do you have difficulty (with self-care such as) washing all over or dressing? a. No – no difficulty

b. Yes – some difficulty c. Yes – a lot of difficulty d. Cannot do at all

1. Using your usual (customary) language, do you have difficulty communicating, for example understanding or being understood?

a. No – no difficulty
b. Yes – some difficulty c. Yes – a lot of difficulty d. Cannot do at all

*Interviewer read:* “Now I am going to ask you some [further] questions about [your/his/her] general mental and physical health. These questions deal with [your/his/her] ability to do different daily activities, as well as with how [you have/he has/she has] been feeling. [Although some of these questions may seem similar to ones you have already answered, it is important that we ask them all.]”

VIS_1

[Do/Does] [you/he/she] wear glasses?

1.Yes
2. No
*7. Refused
9. Don’t know*

VIS_2

[Do/Does] [you/he/she] have difficulty seeing, [*If VIS_1 = 1:* even when wearing [your/his/her] glasses]? Would you say... [*Read response categories*]

1. No difficulty
   2. Some difficulty
   3. A lot of difficulty
   4. Cannot do at all / Unable to do *7. Refused
   9. Don’t know*

*VIS_3*

[Do/does] [you/he/she] have difficulty clearly seeing someone’s face across a room [*If VIS_1 = 1:* even when wearing [your/his/her] glasses]? Would you say... [*Read response categories*]

1. No difficulty
   2. Some difficulty
   3. A lot of difficulty
   4. Cannot do at all / Unable to do *7. Refused
   9. Don’t know*

*VIS_4*

[Do/does] [you/he/she] have difficulty clearly seeing the picture on a coin [*If VIS_1 = 1:* even when wearing [your/his/her] glasses]? Would you say... [*Read response categories*]?

1. 1. No difficulty
   2. Some difficulty
   3. A lot of difficulty
   4. Cannot do at all / Unable to do *7. Refused
   9. Don’t know*

HEAR_1

[Do/Does] [you/he/she] use a hearing aid?

1. Yes
   2. No
   *7. Refused
   9. Don’t know*

HEAR_2

[Do/Does] [you/he/she] have difficulty hearing, [*If HEAR_1 = 1:* even when using a

hearing aid(s)]? Would you say... [*Read response categories*]

1. No difficulty
2. Some difficulty
3. A lot of difficulty
4. Cannot do at all / Unable to do *7. Refused
9. Don’t know*

HEAR_3

How often [do/does] [you/he/she] use [your/his/her] hearing aid(s)? Would you say... [*Read response categories*]

1. All of the time 2. Some of the time 3. Rarely
4. Never
*7. Refused
9. Don’t know*

HEAR_4

[Do/does] [you/he/she] have difficulty hearing what is said in a conversation with one other person in a quiet room [*If HEAR_1 = 1:* even when using [your/his/her] hearing aid(s)]? Would you say... [*Read response categories*]

1. No difficulty
2. Some difficulty
3. A lot of difficulty
4. Cannot do at all / Unable to do *7. Refused
9. Don’t know*

HEAR_5

[Do/does] [you/he/she] have difficulty hearing what is said in a conversation with one other person in a noisier room [*If HEAR_1 = 1:* even when using [your/his/her] hearing aid(s)]? Would you say... [*Read response categories*]

1. No difficulty
2. Some difficulty
3. A lot of difficulty
4. Cannot do at all / Unable to do *7. Refused
9. Don’t know*

MOB_1

[Do/Does] [you/he/she] have difficulty walking or climbing steps? Would you say... [*Read response categories*]

1. No difficulty
2. Some difficulty
3. A lot of difficulty
4. Cannot do at all / Unable to do *7. Refused
9. Don’t know*

MOB_2

[Do/does] [you/he/she] use any equipment or receive help for getting around?

1. Yes
   2. No (*Skip to MOB_4.*)
   *7. Refused* (*Skip to MOB_4.*) *9. Don’t know* (*Skip to MOB_4.*)

MOB_3

[Do/does] [you/he/she] use any of the following?
*Interviewer: Read the following list and record all affirmative responses*:

|  |  | 1. Yes | 2. No | *7. Refused* | *9 Don’t Know* |
| --- | --- | --- | --- | --- | --- |
| A. | Cane or walking stick? |  |  |  |  |
| B. | Walker or Zimmer frame? |  |  |  |  |
| C. | Crutches? |  |  |  |  |
| D. | Wheelchair or scooter? |  |  |  |  |
| E. | Artificial limb (leg/foot)? |  |  |  |  |
| F. | Someone’s assistance? |  |  |  |  |
| G. | Other (please specify): |  |  |  |  |

MOB_4

[Do/Does] [you/he/she] have difficulty walking 100 meters on level ground, that would be about the length of one football field or one city block [*If MOB_2 = 1:* without the use of [your/his/her] aid]? Would you say... [*Read response categories*]

1. No difficulty
2. Some difficulty
3. A lot of difficulty
4. Cannot do at all / Unable to do *(Skip to MOB_6.)*

*7. Refused
9. Don’t know*

[*Note: Allow national equivalents for 100 metres.*]

MOB_5

[Do/Does] [you/he/she] have difficulty walking half a km on level ground, that would be the length of five football fields or five city blocks [*If MOB_2 = 1:* without the use of [your/his/her] aid]? Would you say... [*Read response categories*]

1. No difficulty
2. Some difficulty
3. A lot of difficulty
4. Cannot do at all / Unable to do *7. Refused
9. Don’t know*

[*Note: Allow national equivalents for 500 metres.*]

MOB_6
[Do/Does] [you/he/she] have difficulty walking up or down 12 steps? Would you

say... [*Read response categories*]

1. No difficulty
2. Some difficulty
3. A lot of difficulty
4. Cannot do at all / Unable to do *7. Refused
9. Don’t know*

*If MOB_2 = 2 “No”, skip to next section.
If MOB_3 = D “Wheelchair”, skip to next section.*

COM_1

Using [your/his/her] usual language, [do/does] [you/he/she] have difficulty communicating, for example understanding or being understood? Would you say... [*Read response categories*]

1. No difficulty
2. Some difficulty
3. A lot of difficulty
4. Cannot do at all / Unable to do *7. Refused
9. Don’t know*

COM_2

[Do/does] [you/he/she] use sign language?

1. Yes
   2. No
   *7. Refused
   9. Don’t know*

COG_1

[Do/does] [you/he/she] have difficulty remembering or concentrating? Would you say... [*Read response categories*]

1. No difficulty
2. Some difficulty
3. A lot of difficulty
4. Cannot do at all / Unable to do *7. Refused
9. Don’t know*

*COG_2*

[Do/does] [you/he/she] have difficulty remembering, concentrating, or both? Would you say... [*Read response categories*]

1. Difficulty remembering only
2. Difficulty concentrating only (*skip to next section)* 3. Difficulty with both remembering and concentrating *7. Refused
9. Don’t know*

*COG_3*

How often [do/does] [you/he/she] have difficulty remembering? Would you say... [*Read response categories*]

1. Sometimes
2. Often
3. All of the time *7. Refused
9. Don’t know*

*COG_4*

[Do/does] [you/he/she] have difficulty remembering a few things, a lot of things, or almost everything? Would you say... [*Read response categories*]

1. A few things
   2. A lot of things
   3. Almost everything *7. Refused
   9. Don’t know*

SC_1

[Do/does] [you/he/she] have difficulty with self care, such as washing all over or dressing? Would you say... [*Read response categories*]

1. No difficulty
   2. Some difficulty
   3. A lot of difficulty
   4. Cannot do at all / Unable to do *7. Refused
   9. Don’t know*

UB_1  [Do/Does] [you/he/she] have difficulty raising a 2 liter bottle of water or soda from waist to eye level? Would you say... [*Read response categories*]

1. No difficulty
2. Some difficulty
3. A lot of difficulty
4. Cannot do at all / Unable to do *7. Refused
9. Don’t know*

UB_2  [Do/Does] [you/he/she] have difficulty using [your/his/her] hands and fingers, such as picking up small objects, for example, a button or pencil, or opening or closing containers or bottles? Would you say... [*Read response categories*]

1. No difficulty
2. Some difficulty
3. A lot of difficulty
4. Cannot do at all / Unable to do *7. Refused
9. Don’t know*

*Proxy respondents may be omitted from the next section, at country’s discretion.*

*Interviewer: If respondent asks whether they are to answer about their emotional states after taking mood-regulating medications, say:* “Please answer according to whatever medication [you were/he was/she was] taking.”

ANX_1

How often [do/does] [you/he/she] feel worried, nervous or anxious? Would you say... [*Read response categories*]

1. Daily
2. Weekly
3. Monthly
4. A few times a year 5. Never
*7. Refused
9. Don’t know*

ANX_2

[Do/Does] [you/he/she] take medication for these feelings?

1. Yes
2. No (*If “Never” to ANX_1 and “No” to ANX_2, skip to DEP_1.)*

*7. Refused
9. Don’t know*

ANX_3

Thinking about the last time [you/he/she] felt worried, nervous or anxious, how would [you/he/she] describe the level of these feelings? Would [you/he/she] say... [*Read response categories*]

1. A little
2. A lot
3. Somewhere in between a little and a lot

*7. Refused
9. Don’t know*

9

DEP_1

How often [do/does] [you/he/she] feel depressed? Would [you/he/she] say... [*Read response categories*]

1. Daily
2. Weekly
3. Monthly
4. A few times a year 5. Never
*7. Refused
9. Don’t know*

DEP_2

[Do/Does] [you/he/she] take medication for depression?

1. Yes
2. No (*If “Never” to DEP_1 and “No” to DEP_2, skip to next section.)*

*7. Refused
9. Don’t know*

DEP_4

Thinking about the last time [you/he/she] felt depressed, how depressed did [you/he/she] feel? Would you say... [*Read response categories*]

1. A little
2. A lot
3. Somewhere in between a little and a lot *7. Refused
9. Don’t know*

*Proxy respondents may be omitted from the next section, at country’s discretion.*

*Interviewer: If respondent asks whether they are to answer about their pain when taking their medications, say:* “Please answer according to whatever medication [you were/he was/she was] taking.”

PAIN_1

In the past 3 months, how often did [you/he/she] have pain? Would you say... [*Read response categories*]

1. Never
2. Some days 3. Most days 4. Every day *7. Refused
9. Don’t know*

(*If “Never” to PAIN_1, skip to next section.)*

PAIN_2

Thinking about the last time [you/he/she] had pain, how much pain did [you/he/she] have? Would you say... [*Read response categories*]

1. A little
2. A lot
3. Somewhere in between a little and a lot *7. Refused
9. Don’t know*

TIRED_1

In the past 3 months, how often did [you/he/she] feel very tired or exhausted? Would you say... [*Read response categories*]

1. Never
2. Some days 3. Most days 4. Every day *7. Refused
9. Don’t know*

(*If “Never” to TIRED_1, skip section.)*

TIRED_2

Thinking about the last time [you/he/she] felt very tired or exhausted, how long did it last? Would you say... [*Read response categories*]

1. Some of the day 2. Most of the day 3. All of the day *7. Refused*

*9. Don’t know*

TIRED_3

Thinking about the last time [you/he/she] felt this way, how would you describe the level of tiredness? Would you say... [*Read response categories*]

1. A little
2. A lot
3. Somewhere in between a little and a lot *7. Refused
9. Don’t know*

End of Questionnaire.
